# Histone deacetylase 6 as a novel promising target to treat cardiovascular disease

**DOI:** 10.1002/cai2.114

**Published:** 2024-05-07

**Authors:** Ya‐Xi Wu, Bing‐Qian Li, Xiao‐Qian Yu, Yu‐Lin Liu, Rui‐Hao Chui, Kai Sun, Dian‐Guang Geng, Li‐Ying Ma

**Affiliations:** ^1^ State Key Laboratory of Esophageal Cancer Prevention and Treatment, Key Laboratory of Advanced Pharmaceutical Technology, Ministry of Education of China, School of Pharmaceutical Science and Institute of Pharmaceutical Science Zhengzhou University Zhengzhou Henan China; ^2^ Key Laboratory of Cardio‐Cerebrovascular Drugs' China Meheco Topfond Pharmaceutical Co. Zhumadian Henan China

**Keywords:** cardiovascular disease, histone deacetylase 6, inhibitor

## Abstract

Histone deacetylase 6 (HDAC6) belongs to a class of epigenetic targets that have been found to be a key protein in the association between tumors and cardiovascular disease. Recent studies have focused on the crucial role of HDAC6 in regulating cardiovascular diseases such as atherosclerosis, myocardial infarction, myocardial hypertrophy, myocardial fibrosis, hypertension, pulmonary hypertension, and arrhythmia. Here, we review the association between HDAC6 and cardiovascular disease, the research progress of HDAC6 inhibitors in the treatment of cardiovascular disease, and discuss the feasibility of combining HDAC6 inhibitors with other therapeutic agents to treat cardiovascular disease.

## Graphical abstract

HDAC6 have been found to be a key protein in the tumor–cardiovascular association. HDAC6 inhibitors may have important moderating effects in various types of cardiovascular diseases. HDAC6 inhibitors alone or in combination with other agents have shown promising prospects in the treatment of cardiovascular disease.

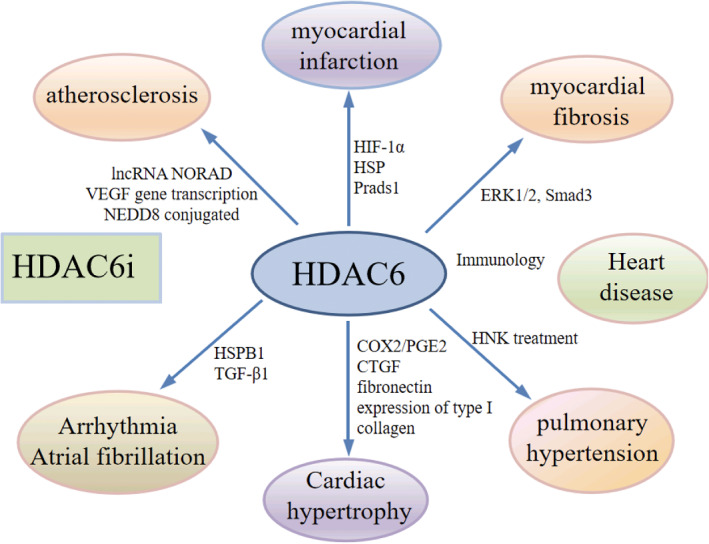

**Abbreviations**
AF: atrial fibrillation
Ang‐II: angiotensin II
ANP: atrialnatriureticpeptide
CD1/2: catalytic domain 1/2
CRP: C‐reactive protein
CSE: cystathionine‐γ‐lyase
CVF: collagen volume fraction
FGFRs: fibroblast growth factor receptors
HDAC6: histone deacetylase 6
HDACs: histone deacetylases
HF: heart failure
HNK: honokiol
HSP90: heat shock protein 90
LVEDP: left ventricular end‐diastolic pressure
MCT: medium‐chain triglycerides
MeCP2: methyl‐CpG binding protein 2
MF: myocardial fibrosis
MI: myocardial infarction
MIRI: myocardial ischemia‐reperfusion injury
PASMC: pulmonary artery smooth muscle cells
PPAR: peroxisome proliferator activated receptor
Prx: peroxiredoxin
ROS: reactive oxygen species
TGF‐β: transforming growth factor‐β
TNF‐α: tumor necrosis factor‐α
T‐PA: tissue plasminogen activator

## 1 BACKGROUND

With the development of medical and health undertakings, improvements in tumor diagnosis and treatment have significantly prolonged patient survival. However, complications associated with ongoing drug treatment, particularly cardiovascular toxicity, have become the leading cause of death for patients [1, 2, 3, 4, 5, 6, 7]. Consequently, exploring the regulatory mechanisms linking tumor progression and cardiovascular injury and developing drugs with high efficiency, low cardiovascular toxicity, and even cardioprotective effects are urgent issues in tumor cardiology.

Lysine acetylation is a reversible posttranslational modification. Many studies have shown that histone deacetylases (HDAC) play a significant role in the regulatory mechanisms between tumors and cardiovascular disease [8, 9, 10, 11]. So far, mammalian HDAC can be mainly divided into four types according to structure, cell location, and enzyme activity (Table 1) [12, 13, 14]. Class I HDAC (HDAC1, 2, 3, and 8) are widely expressed in the nucleus. They contain a catalytic functional domain surrounded by N‐ and C‐terminal regions. Class II HDAC enzymes are comprised of two small groups that are characterized by shuttling activity between the nucleus and cytoplasm. They include group IIa (HDAC4, 5, 7, and 9) and group IIb (HDAC6 and 10), and the enzymes contain one or two catalytic sites [15]. Class III enzymes are the sirtuins (1–7), which are a class of NAD+dependent HDACs and ADP ribosyltransferases. Silencing information regulators (SIRT)−1 and 6–7 are mainly located in the nucleus, SIRT2 is located in the cytoplasm, and SIRT3–5 are located in mitochondria [16]. Class IV HDAC consists of a single enzyme, HDAC11, which is located in the nucleus and is homologous to the yeast proteins RPD3 and HDA1 [17].

**Table 1:** Classification, localization, and enzyme activities of histone deacetylases.

Classification	Members	Localization	Enzyme activities
I	HDAC1	Nucleus	Deacetylation
	HDAC2	Nucleus	Deacetylation
	HDAC3	Nucleus	Deacetylation, Decrotonylation
	HDAC8	Nucleus	Deacetylation
IIa	HDAC4	Nucleus and cytoplasm	Deacetylation
	HDAC5	Nucleus and cytoplasm	Deacetylation
	HDAC7	Nucleus and cytoplasm	Deacetylation
	HDAC9	Nucleus and cytoplasm	Deacetylation
IIb	HDAC6	Nucleus	Deacetylation
	HDAC10	Cytoplasm	Deacetylation
III	SIRT1	Nucleus	Deacetylation
	SIRT2	Cytoplasm	Deacetylation
	SIRT3	Mitochondria	Deacetylation
	SIRT4	Mitochondria	ADP‐ribosylation
	SIRT5	Mitochondria	Desuccinylation and demalonylation
	SIRT6	Nucleus	ADP‐ribosylation, deacetylation
	SIRT7	Nucleus	Deacetylation
IV	HDAC11	Nucleus	Deacetylation

Abbreviations: HDAC, histone deacetylase; SIRT, silent information regulator.

Of the zinc‐dependent HDACs, HDAC6 has a variety of biological functions because of its unique protein structure and diverse substrate types [18]. Its abnormally high expression in cells is closely related to the development of many diseases. These include neurodegenerative diseases, cancer, cardiovascular diseases, autoimmune diseases, and others [19, 20, 21]. Current studies show that HDAC6 can promote the occurrence and development of cardiovascular disease and related tumors. While inhibiting HDAC6 activity can effectively prevent tumor progression and cardiovascular damage, representing a regulatory mechanism between tumors and cardiovascular disease. There are currently many reviews describing the relationship between HDAC6 and the regulation of tumors [12, 22, 23, 24]. However, there are few reports on the relationship between HDAC6 and its pathogenesis and regulatory function in cardiovascular disease. Therefore, in this article, we will review the association between HDAC6 and cardiovascular disease, describe research progress on HDAC6 inhibitors for the treatment of cardiovascular disease, and discuss the feasibility of combining HDAC6 inhibitors with other therapeutic agents to treat cardiovascular disease.

## 2 STRUCTURE AND BIOLOGICAL FUNCTION OF HISTONE DEACETYLASE 6 (HDAC6)

### 2.1 Structure of HDAC6

**Figure 1:**
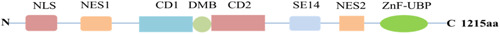
HDAC6 domain structure.

HDAC6 is a IIb cytoplasmic deacetylase, consisting of 1215 amino acids, which was widely documented in 1990 [20, 25]. It is unique among HDACs, having two highly conserved catalytic domains [15] (CD1 and CD2) and a C‐terminal zinc finger ubiquitin‐binding domain (ZNF‐UBP) [25, 26, 27, 28]. HDAC6 also contains nuclear export signaling (NES) sequences (NES1 and NES2) and a cytoplasmic anchoring domain (SE14) (Figure 1) [26, 27], as shown in Figure 1. The NES allows HDAC6 to shuttle freely between the nucleus and cytoplasm, while SE14 stably retains HDAC6 in the cytoplasm. Through NES and SE14, HDAC6 is mainly localized in the cytoplasm [29, 30, 31]. The ZNF‐UBP domain binds to free ubiquitinated, monoubiquitinated, and polyubiquitinated proteins. Through this domain, HDAC6 plays a key role in the clearance of intracellular misfolded proteins [26, 32].

### 2.2 Biological function of HDAC6

Overall, HDAC6 can catalyze histone deacetylation, enable nucleosome compaction, and inhibit gene transcription and translation. In addition, HDAC6 can act on nonhistone substrates in the cytoplasm, such as α‐tubulin [29], β‐catenin [30], tau protein [31], heat shock protein (HSP)−90 [32], peroxiredoxin (Prdx) [33], and cortactin [34]. Thus, HDAC6 has far‐reaching biological functions [35].

## 3 THE REGULATORY RELATIONSHIP BETWEEN HDAC6 AND CARDIOVASCULAR DISEASE

According to global research reports, HDAC6 plays an essential regulatory role in cardiovascular disease, being associated with the occurrence and development of myocardial infarction (MI), myocardial fibrosis (MF), cardiac hypertrophy, pulmonary hypertension, arrhythmia, and atherosclerosis. Here, we will expand upon these six common cardiovascular diseases.

### 3.1 Myocardial infarction

The leading cause of MI is thrombosis in the coronary artery. If thrombosis continues to block the coronary artery, the time of myocardial ischemia will be prolonged and irreversible damage occurs in the myocardium. Meanwhile, tissue plasminogen activator is rapidly released near the plaque to dissolve the local thrombus. Acute MI is a dangerous and critical condition in the clinic, characterized by acute myocardial tissue, continuous ischemia, and hypoxia. At present, the most widely used therapeutic strategy is to restore blood supply to the ischemic myocardium. However, restoration of blood flow will cause further damage to the internal structure and function of the myocardium, including expansion of infarct size, ventricular dysfunction, and post‐MI cardiac fibrosis [36]. These can unfortunately offset the benefits of therapy and are defined as myocardial ischemia‐reperfusion injury [33].

**Figure 2:**
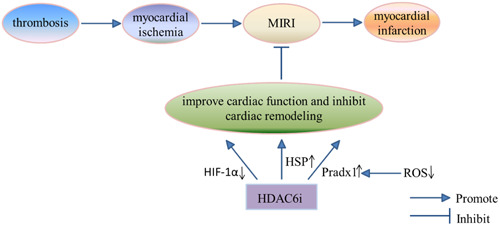
The regulatory mechanism of HDAC6 and myocardial infarction. HDAC6i, HDAC6 inhibitor.

At present, HDAC6 inhibitors can potentially reduce ischemia/reperfusion (IR) injury, improve cardiac function, and reduce infarct size (Figure 2). Lin et al. [34] examined a cardiac IR model of left coronary artery ligation reperfusion in male Wistar rats. They found that regulating hypoxia‐inducible factor 1α (HIF‐1α) inhibited HDAC6, and ultimately reduced infarct size in rats with cardiac IR injury. Transforming growth factor‐β (TGF‐β) and C‐reactive protein can be useful biomarkers to monitor the efficacy of HDAC6 inhibitors in cardiac IR injury. Furthermore, using an in vitro heart model, Leng and colleagues [35] showed that HDAC6 inhibition can improve cardiac function, attenuate reactive oxygen species generation, reduce cardiac infarction, and increase acetylated‐Prdx1 levels (by regulating Prdx1 acetylation at K197) after MI/R or hypoxia/reperfusion injury. In addition, HDAC6 inhibition improved cardiac function, weakened the effect of heart failure on fibroblast growth factor receptors, peroxisome proliferator activated receptor subtypes, and pro‐inflammatory cytokines, shortened cardiac ejection fraction, and improved ventricular size [36]. Currently, the most significant advantage of HDAC6 inhibitors in protecting the myocardium against IR injury is the maintenance of normal protein homeostasis and prevention of aggregation and accumulation of faulty proteins. Nagata et al. [37] found that inhibition of HDAC6 activity enhanced translocation of heat shock transcription factor 1 into the nucleus, enhanced induction of HSP expression, and ultimately sustained a prolonged and stable cell protein state. HDAC6 inhibition also improved cardiac pumping function after MI. Zhang et al. [38] found that administration of HDAC6 inhibitor for 2 months significantly reduced cardiac dysfunction, promoted cardiac repair, and inhibited cardiac remodeling. Therefore, HDAC6 can both improve cardiac function after MI and inhibit cardiac remodeling. In the long term, inhibition of HDAC6 activity can effectively treat heart failure after MI.

### 3.2 Myocardial fibrosis

**Figure 3:**
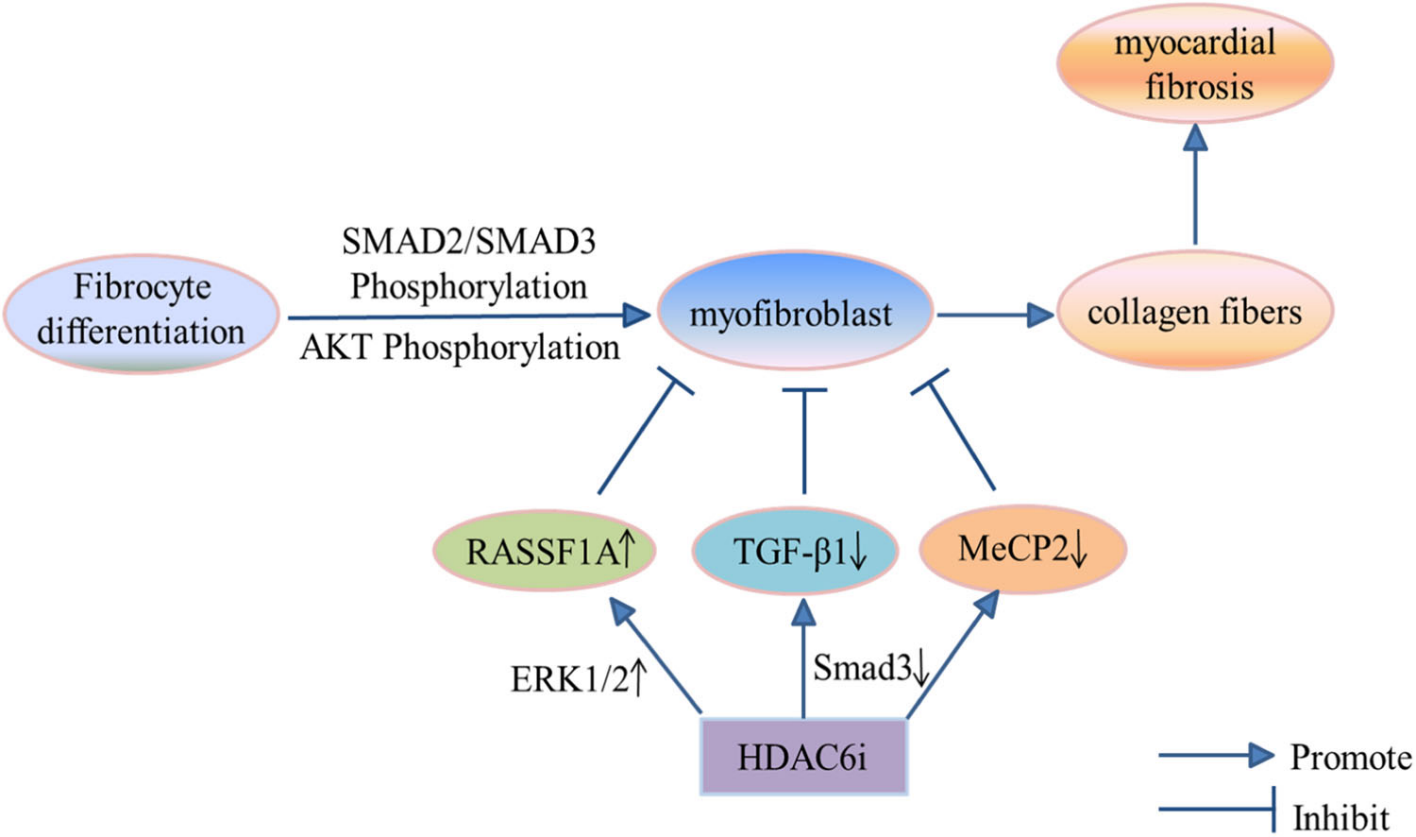
The regulatory mechanism of HDAC6 and myocardial fibrosis. HDAC6i, HDAC6 inhibitor.

Excessive accumulation of collagen fibers in myocardial tissue is called MF and results in a significant increase in collagen concentration or collagen volume fraction. In recent years, accumulating studies have shown that various cardiovascular diseases (including hypertension, heart failure, and MI) are related to MF. It can be viewed as a standard pathological change of various heart diseases at a particular stage. It is one of the main manifestations of myocardial remodeling, which can lead to increased myocardial stiffness, decreased ventricular diastolic function, decreased coronary artery reserve, and even sudden death. However, HDAC6 inhibitors can significantly inhibit fibrosis [39], as shown in Figure 3. Tao et al. [39] demonstrated that HDAC6 inhibition using the inhibitor, tubacin, or a small interfering (si)RNA ultimately attenuated production of TGF‐β1‐induced myoblast markers. In contrast, siRNA knockdown of HDAC6 inhibited cardiac fibroblast proliferation. They also found that HDAC6 knockdown increased the expression of Ras association domain family 1 isoform A (RASSF1A) in activated cardiac fibroblasts. RASSF1A may affect activation of cardiac fibroblasts by influencing the extracellular signal‐regulated kinase 1/2 (ERK1/2) signaling pathway, thereby controlling the proliferation and fibrosis of cardiac fibroblasts. Tao et al. [28] found that HDAC6 inhibitor and HDAC6 siRNA were effective in treating cardiac fibroblasts, completely restoring acetylation levels of α‐tubulin and preventing cell proliferation. Perhaps through HDAC6, methyl‐CpG binding protein 2 (MeCP2) negatively controls acetylation of α‐tubulin in cardiac fibroblast proliferation and fibrosis. In addition, acetylation of type I collagen is regulated by HDAC6/p300 acetyltransferase, which was first demonstrated by Choi [40]. The results of chromatin immunoprecipitation analyses showed that TGF‐β induced acetylated histone H4 or phospho‐Smad2/3 binding elements to Smad3 in promoters of fibrosis‐related genes (including collagen type I), which were prevented by HDAC6 inhibitors. The mechanism may be that HDAC6 inhibitors and Smad3 downregulation synergistically blocks the function of TGF‐β or angiotensin II (Ang II) in fibrosis. These findings suggest that HDAC6 plays an essential regulatory role in controlling the pathological process of MF, and thereby provides a new therapeutic strategy for MF.

### 3.3 Cardiac hypertrophy

**Figure 4:**
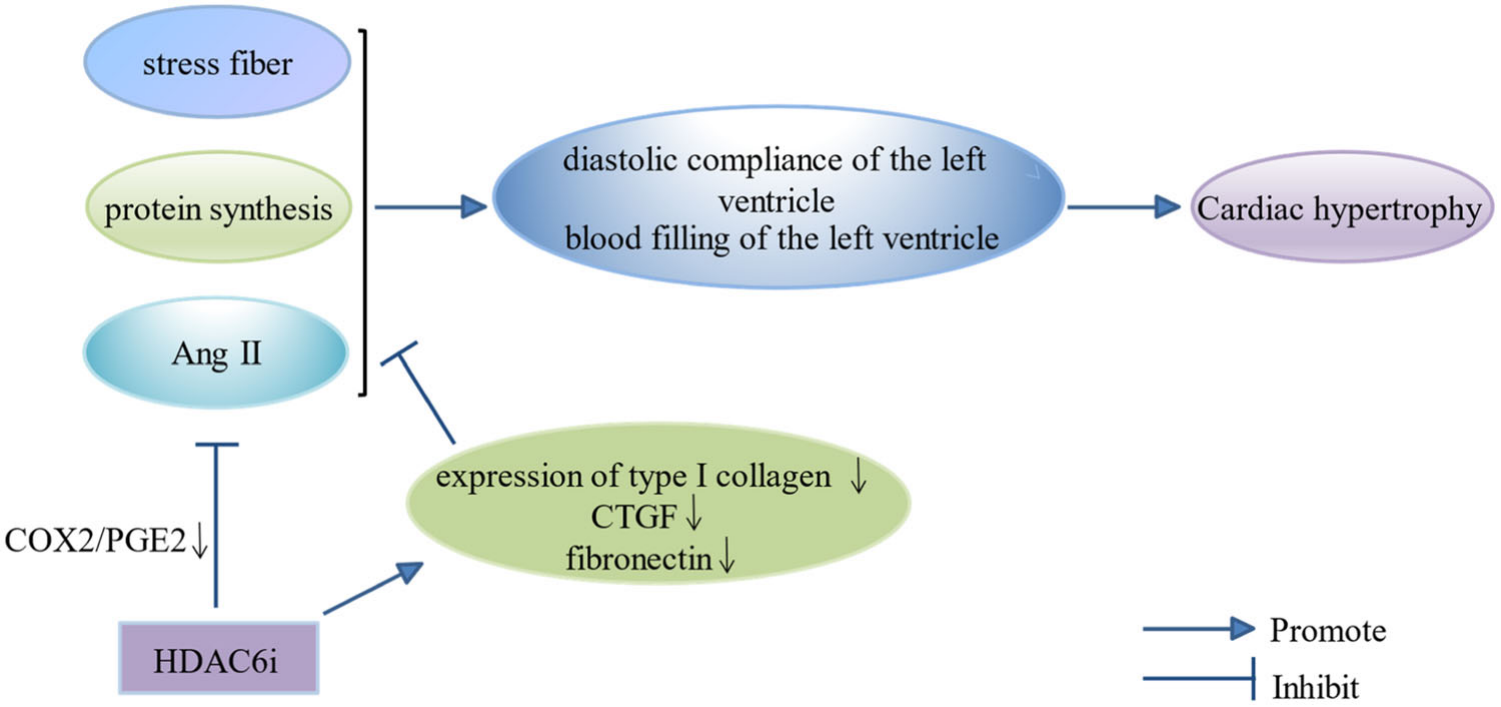
The regulatory mechanism of HDAC6 and cardiac hypertrophy. HDAC6i, HDAC6 inhibitor.

Compensatory disorders, arrhythmias, sudden death, and dilated cardiomyopathy are all associated with heart fat and lead to heart failure that can be described as heart hypertrophy; that is, enlargement of the heart in response to physiological or pathological stimuli [41]. Cardiac hypertrophy is associated with reactivation of fetal gene expression, increased stress fiber formation, and increased protein synthesis. It is defined by symmetric ventricular wall hypertrophy, and often with intrusions into the ventricular septum, small ventricles, left ventricular congestion blocked, and decreased diastolic compliance of the left ventricle. The role of HDAC6 in cardiac hypertrophy has been widely studied (Figure 4) [41, 42]. In a mouse model of deoxycorticosterone acetate (DOCA) salt hypertension‐induced cardiac hypertrophy, Kee et al. [41] found that HDAC6 inhibition regulates cardiac hypertrophy through the activities of HDAC enzymes such as HDAC6 and HDAC8. It also prevented cardiac fibrosis in DOCA saline hypertensive rats by downregulating the expression of type I collagen, connective tissue growth factor, and fibronectin. Furthermore, Zhang et al. [42] demonstrated the effect of a HDAC6 inhibitor on Ang II‐induced cardiac hypertrophy using an in vitro myocardial H9C2 cell model. HDAC6 inhibitor prevented cyclooxygenase 2 (COX2)/prostaglandin E2 (PGE2) pathway activation in a HDAC5/HDAC6‐dependent manner; this is thought to be the mechanism that ultimately alleviates Ang II‐induced myocardial hypertrophy. Therefore, HDAC6 is a promising therapeutic target for pathologic heart hypertrophy.

### 3.4 Heart disease and pulmonary hypertension

Hypertension has been gaining attention as one of the main risk factors for the development of systemic vascular disease and cardiovascular disease. Pulmonary hypertension is a progressive disease characterized by right heart failure and pulmonary vascular remodeling, both of which are serious threats to human health. The role of HDAC6 in cardiac and skeletal muscle remodeling, induced by Ang II, plays a central role in blood pressure control, heart failure, and associated skeletal muscle depletion [43]. Resistance of HDAC6 inhibitors to chronic Ang II signaling‐mediated skeletal muscle depletion was reported by Demos‐Davies et al. [43]. These findings provided a new role of HDAC6 in striated muscle, highlighting the potential of HDAC6 selective inhibitors for the treatment of cardiac dysfunction and muscle atrophy in patients with heart failure. Habibian et al. [44] suggested a role of HDACs and HDAC inhibitors as regulators of acetyl‐phosphorylation crosstalk in the control of cardiac function. Lee et al. [45] found that inhibiting HDAC6 reduced transcriptional activity of salt corticosteroid receptors through acetylation, thus preventing the progression of hypertension. In addition, dysregulation of vascular smooth muscle cell contraction (especially constrictive overextension leading to vascular smooth muscle cell enlargement) leads to hypertension [46]. Furthermore, Chi et al. [47] suggested that honokiol treatment protects cystathionine γ‐lyase against HDAC6‐mediated degradation and may constitute an alternative for preventing endothelial dysfunction and hypertensive disorders.

**Figure 5:**
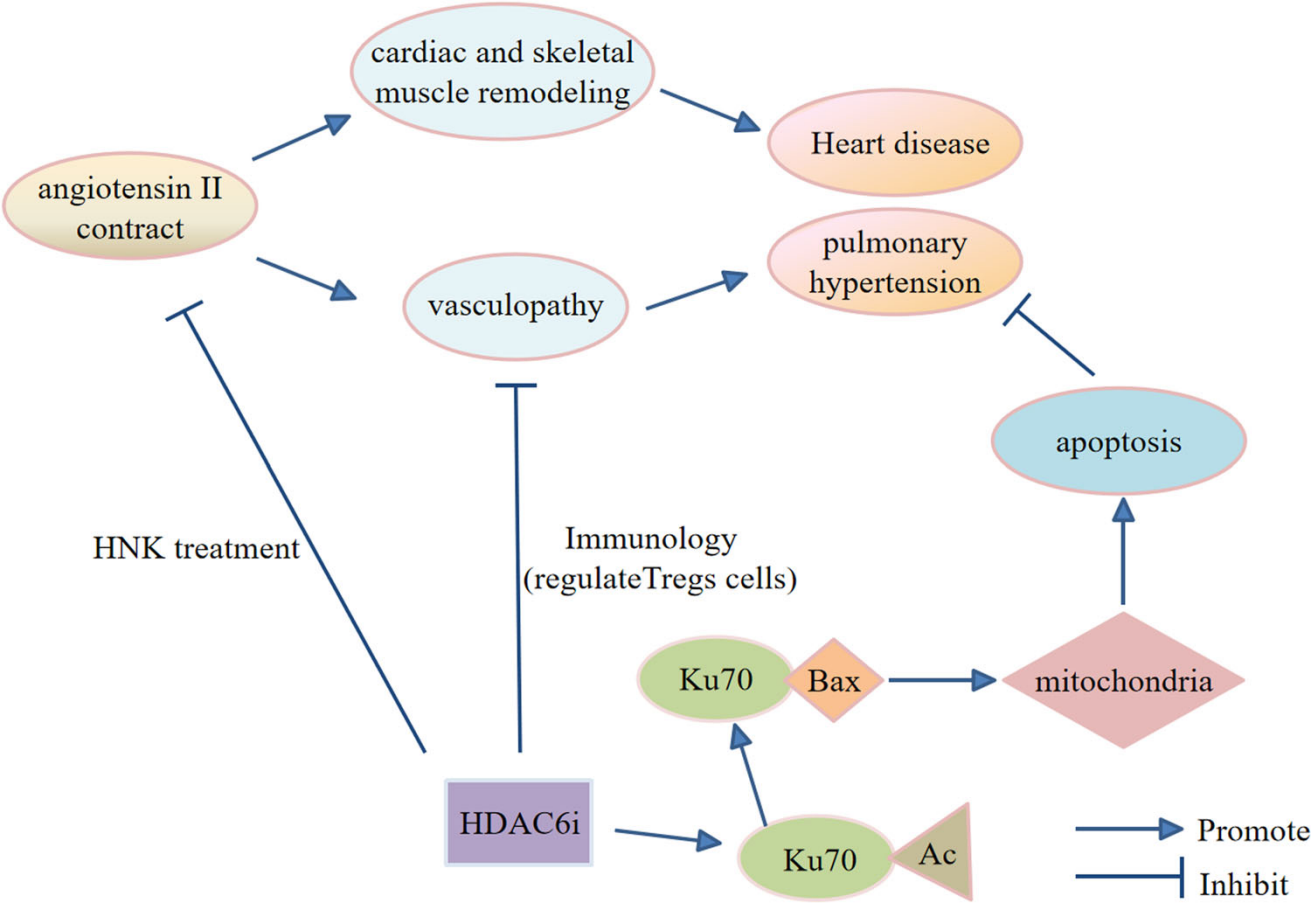
The regulatory mechanism of HDAC6 and heart disease and pulmonary hypertension. HDAC6i, HDAC6 inhibitor.

Zhao et al. [48] found that increased HDAC activity contributes to the vascular pathology of pulmonary hypertension. The effectiveness of HDAC6 inhibitors in pulmonary hypertension models supports this treatment strategy for reducing pulmonary hypertension (Figure 5). In recent years, the study of pulmonary hypertension has become increasingly related to immunology. Previous studies have found that pulmonary hypertension is more likely to occur in experimental mice without a thymus (T cell deficiency) [49]. Other studies have found that regulatory cells [50] (CD4+, CD25+, and FoxP3+) can block progression of vascular injury and resist formation of pulmonary hypertension. Acetylation and deacetylation complexes exist in regulatory T cells (Tregs). Their function is regulated by HDAC, suggesting that HDAC inhibitors may inhibit pulmonary hypertension by regulating Tregs function. In addition, Boucherat et al. [51] found that HDAC6 was significantly upregulated in the lung, distal prealbumin, and pulmonary artery smooth muscle cells (PASMCs) isolated from pulmonary arterial hypertension (PAH) patients and animal models. The mechanism might be that HDAC6 maintains Ku70 (as a nuclear protein) in a low acetylation state, blocking translocation of Bax to mitochondria, and successfully preventing cell apoptosis. Inhibition of HDAC6 had a partial protective effect on chronic hypoxia‐induced pulmonary hypertension. In conclusion, the administration of HDAC6 inhibitors may be a potential therapeutic agent for treating pathological heart disease and pulmonary hypertension, regulatory Trges cells are a specialized subpopulation of T cells that act to suppress immune respone, thereby maintaining homeostasis and self‐tolerance.

### 3.5 Arrhythmia

**Figure 6:**
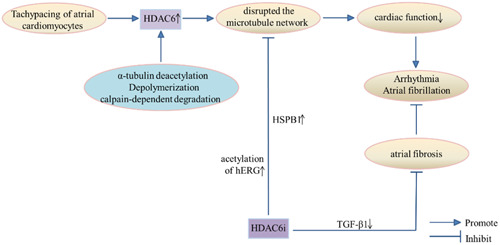
The regulatory mechanism of HDAC6 and arrhythmia. HDAC6i, HDAC6 inhibitor.

Abnormal excitation in or outside the sinoatrial node is known as arrhythmia, and often occurs with slow, blocked, or abnormal conduction of excitation through the cardiac pathway. This means that the origin of cardiac activity and/or conduction disorders is a cause of abnormal arrhythmia (or frequency). The arrhythmias are an important group of cardiovascular diseases. The most common, persistent clinical tachyarrhythmia is known as atrial fibrillation (AF). This is a significant contributor to cardiovascular morbidity and mortality, and AF is progressive in nature because atrial remodeling provides a further basis for arrhythmia. Due to difficulties of current medications in addressing dilatation of AF‐affected stroma, conversion to a sinus rhythm is less likely as the arrhythmia continues [52]. Therefore, other measures are necessary to limit or reverse the cellular matrix affected by AF and the molecular changes that lead to structural remodeling and drive the onset and progression of arrhythmias. Importantly, during the pathogenesis of several diseases, chromatin packaging is largely determined by the acetylated state of histone 6, which is controlled by histone acetyltransferase and HDAC. Evidence for epigenetic regulation of AF has been demonstrated by examining this epigenetic pathway, whereby the (re)‐activation of fetal genetic programs in cardiomyocytes promotes AF [52]. In addition, transgenic mice with increased HDAC activity are prone to atrial arrhythmias and known substrates of AF, such as myocardial hypertrophy, decreased connexin 40 expression, and fibrosis. Tachypacing of atrial cardiomyocytes increased HDAC6 expression and activity, which disrupted the microtubule network through α‐tubulin deacetylation, depolymerization, and calpain‐dependent degradation. This ultimately severely impaired cardiac function [52, 53]. In conclusion, HDAC6 substantially affects cardiomyocyte protein stability and is involved in the progression of AF (Figure 6).

Zhang et al. [54] found that HDAC6 inhibitor was protected by disruption of α‐tubulin homeostasis and electrical remodeling induced by atrial tachycardia. This improved cellular Ca2+ processing, contractile function, and effectively prevented the progression of AF. Sawa et al. [55] showed that chronic activation of cardiac HDAC6 caused atrial electrical and structural remodeling in an isolated mouse heart model, leading to persistent AF. Cardiac HDAC6 catalytic activity may play an important role in the development of AF induced by hypertension. Hu et al. [56] also found that GGA*‐59 (as heat shock protein inducer) and recombinant HSPB1 are induced by HSP, and accelerate recovery from total protein (TP)‐induced structural remodeling and contractile dysfunction in HL‐1 cardiomyocytes. GGA*‐59 increased HSPB1 levels, restored contractile protein and microtubule levels, and inhibited HDAC6 activity after TP administration. Li et al. [57] found that the stability of mutant human ether‐a‐go‐go related gene (hERG) (as a voltage‐gated potassium ion channel) could be restored by inhibiting HDAC6, possibly indicating that hERG is a substrate for HDAC6. Inhibition of HDAC6‐induced acetylation of hERG reduced ubiquitination, causing hERG stabilization and inhibition of arrhythmias. Recent studies have also shown that HDAC6 is elevated in cardiac fibrous tissue, and TGF‐β1‐induced proliferation of myofibroblasts is reduced by knockout of HDAC6 [39]. However, TGF‐β1 overexpression can cause selective atrial fibrosis and AF [40]. This means that TGF‐β1, as a mediator of atrial fibrosis, inhibits HDAC6 and subsequently downregulates TGF‐β1 expression, thereby reducing atrial fibrosis and preventing AF. Thus, there is strong evidence that HDAC6 leads to imbalanced protein homeostasis, an impaired microtubule system, and reduced transient calcium channels by deacetylating α‐tubulin and initiating development of AF. This suggests that inhibition of HDAC6 could be a targeted therapeutic strategy for clinical AF [52, 54].

### 3.6 Atherosclerosis

Atherosclerosis starts with endothelial cell dysfunction and lipid accumulation in the blood vessel wall, which leads to lipostria lesions [58]. Atherosclerosis describes a group of the most common and important vascular diseases. Typical characteristics of arteriosclerosis include thickening and hardening of the arterial wall, loss of elasticity, and narrowing of the lumen. Atherosclerosis is a feature of lesions arising from the lining of affected arteries. In combination with multiple lesions (including local lipids), there is a gradually degenerating middle artery, fibrous tissue hyperplasia, calcinosis plaque formation, complex carbohydrate accumulation, secondary hemorrhagic lesions with plaques, plaque rupture, and thrombosis. Previous studies have shown that increased HDAC6 activity is a major factor in oxidized low‐density lipoprotein (oxLDL)‐induced endothelial dysfunction in vitro [59]. In endothelial cells, oxLDL can effectively enhance the production of pro‐inflammatory cytokines such as interleukin (IL‐6), IL‐1β, tumor necrosis factor‐α (TNF‐α), and adhesion molecules (by altering adhesion molecule expression on the cell surface), ultimately inducing a pro‐atherogenic effect. Furthermore, increased HDAC6 activity downregulates expression of endothelial cystathionine lyase‐γ. Atherosclerosis has long been considered a chronic inflammatory disease [60], and HDAC2 and HDAC6 may induce early atherosclerotic lesions by promoting the activation, recruitment, adhesion, and migration of white blood cells to the intima.

**Figure 7:**
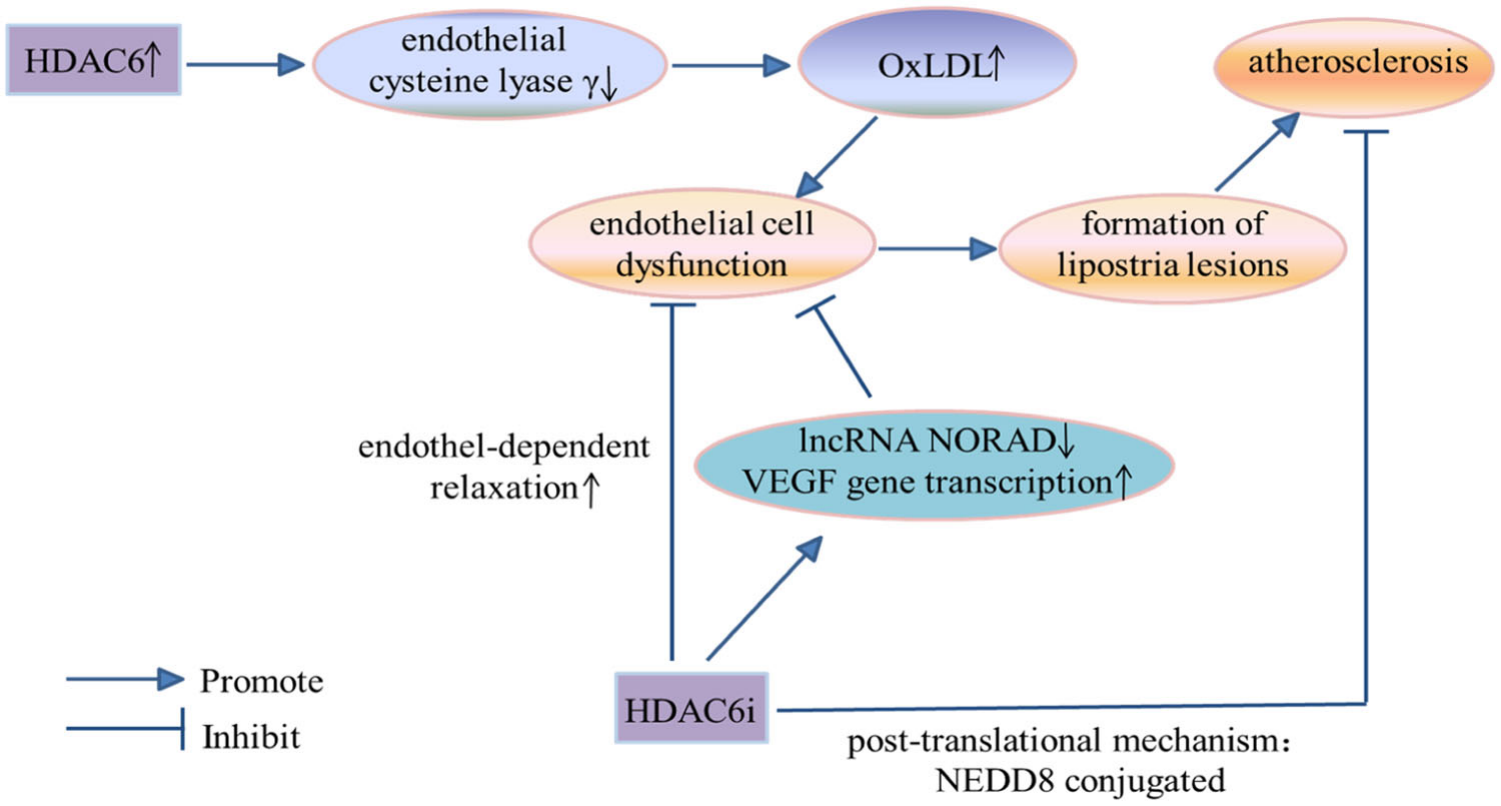
The regulatory mechanism of HDAC6 and atherosclerosis. HDAC6i, HDAC6 inhibitor.

HDAC6 inhibitors are an effective way to treat atherosclerosis (Figure 7). Nomura et al. [59] found that HDAC6 inhibitors restored endothelial‐dependent relaxation, prevented the development of atherosclerosis, and significantly reduced plaque burden in atherosclerotic mouse models. Meanwhile, they also identified a novel posttranslational mechanism that effectively controlled HDAC6 activity in the presence of oxidative damage. Specifically, NEDD8 (as a ubiquitin protein that can modify the substrate protein) conjugated to lysine in the ubiquitin‐binding domain at the C‐terminus of HDAC6 to exert an antiatherogenic effect. Kai et al. [61] found that expression of long noncoding (lnc)RNA NORAD was increased in thoracic aorta of ox‐LDL‐treated human umbilical vein endothelial cells (HUVECs) and atherosclerotic mice. Importantly, lncRNA NORAD knockdown in vitro and in vivo effectively reduced vascular endothelial cell injury and atherosclerosis. LncRNA‐NORAD recruited HDAC6 to enhance H3K9 deacetylation and inhibit vascular endothelial growth factor gene transcription, thereby enhancing vascular endothelial cell damage and atherosclerosis. Therefore, HDAC6 has utility as a diagnostic marker and therapeutic target for atherosclerosis.

## 4 HDAC6 INHIBITORS INVOLVED IN CARDIOVASCULAR DISEASE

Many recent studies have focused on the crucial role of HDAC6 in regulating cardiovascular disease. Inhibition of HDAC6 can reverse the occurrence and progression of cardiovascular disease. Based upon structural characteristics, HDAC6 inhibitors with potential therapeutic effects on cardiovascular disease can be divided into three categories: hydroxamic acids, short‐chain fatty acids, and benzamides.

### 4.1 Pan‐inhibitors

#### 4.1.1 Hydroxamic acids and benzamides

**Figure 8:**
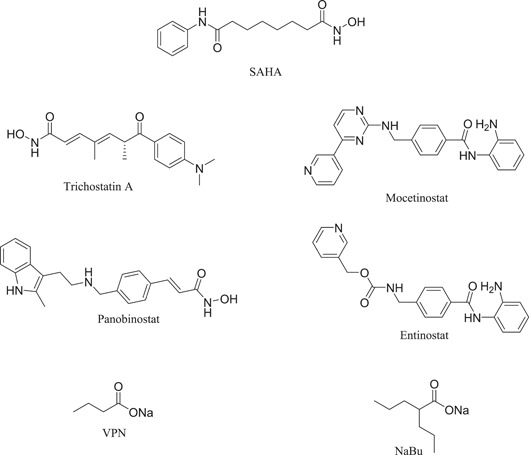
Pan‐inhibitors.

The majority of hydroxamic acids and benzamides used for cardiovascular disease are pan‐inhibitors, including vorinostat, trichostatin A mocetinostat, panobinostat, and entinostat (Figure 8). Among them, vorinostat, trichostatin A, and mocetinostat are highly promising for the treatment of cardiovascular disease [8, 9, 62, 63, 64, 65, 66]. Recently, Lin et al. [66] showed that vorinostat had a powerful antihypertrophic effect, improving diastolic function, reducing left ventricular end‐diastolic pressure (LVEDP), and ultimately improving lung compliance and function. In ApoE knockout mice, Zúñiga‐Muñoz et al. and Manea et al. [67, 68] found that NADPH oxidase and TNF‐α expression were reduced by vorinostat, which mitigated the occurrence of oxidative damage and inflammation and slowed the progression of aortic atheromatous lesions. They also demonstrated that vorinostat improved diastolic function and restored impaired myofibrillary relaxation by reducing LVEDP and the linear period of muscle fiber relaxation in cats with heart failure due to pressure overload. In addition, vorinostat induced metabolic effects, thereby restoring energy supply to the failing heart. Vorinostat was also shown to improve the ability of reparative M2 macrophages and decrease the ability of pro‐inflammatory M1 macrophages in the myocardial infarct area. As a result, the expression of pro‐inflammatory genes was decreased (IL‐1B, IL‐6, and TNF), and that of anti‐inflammatory genes was increased (IL‐4 and IL‐10) [68]. In addition, vorinostat promotes the early and robust recruitment of CD45+/CD11b+/CD206+ (M2) reparative macrophage and indirectly appear to exert a cardioprotective effect [69].

Pan‐inhibitor prevention of cardiac fibrosis has been examined in preclinical animal models. The degree of fibrosis was significantly lower in transverse aortic constriction‐induced MF mice treated with trichostatin A compared with untreated controls [11, 70]. In addition,  Xie et al. [71] found that trichostatin A pretreatment recovered ventricular function and reduced infarct size in MI rats. Furthermore, cardiac hypertrophy caused by the transverse aortic bundle was alleviated by trichostatin A [67, 72]. The expression of actin, collagen, and fibronectin of α‐smooth muscle (essential proteins for myofibroblast proliferation) were reduced by trichostatin A and mocetinostat, resulting in inhibition of transdifferentiation of fibroblasts into myofibroblasts [73, 74]. In cardiomyocytes, glucose‐mediated insulin‐like growth factor 1 receptor was inhibited by treatment with trichostatin A, showing that histone acetylation modulates cardiac hyperglycemia [11].

#### 4.1.2 Short‐chain fatty acids

Short‐chain fatty acid HDAC6 inhibitors are usually in the form of carboxylic acids and have been shown to have low activity in the metal‐binding region of HDAC6. First, valproic acid significantly reduced myocardial hypertrophy caused by long‐term Ang II infusion or aortic ligation in mice or rats [55]. Moreover, valproic acid and other HDAC inhibitors reduced the development of α‐tubulin and β‐myosin heavy chain and interstitial fibrosis [55]. Collagen deposition, upregulation of profibrotic and pro‐inflammatory genes, and macrophage infiltration into the kidney were all attenuated by valproic acid in a mouse model of doxorubicin (Dox)‐induced nephropathy [55]. Valproic acid can also affect lung endothelial cells and vascular smooth muscle cells, reducing pulmonary vascular remodeling, and thereby reducing pulmonary hypertension beyond their anti‐inflammatory, antifibrotic, and antithrombotic properties [41]. In addition, valproic acid treatment significantly reduced cardiac damage after MI, and this cardioprotective effect was mediated by the FOXM1 pathway [63, 73]. Sodium butyrate and entinostat significantly decreased nitric oxide synthase 3 mRNA levels in HUVECs 36 h after angiogenic suppressive treatment [35]. Meanwhile, Zhang and colleagues [42] reported that COX2/PGE2 expression was inhibited by the HDAC inhibitor, sodium butyrate (NaBu). Production of atrial natriuretic peptide and phosphorylated ERK can be stimulated by Ang II and reversed by NaBu in vivo and in vitro.

### 4.2 Selective inhibitors

So far, specific HDAC6 inhibitors have been found to treat cardiovascular disease, including ACY‐1215, ACY‐241, tubacin, tubastatin A, and nextrastat A [1, 15, 18, 55, 66, 75]. Recently, Lin et al. [34] indicated that ACY1215 can regulate the expression of HIF‐1α to reduce infarct size after cardiac IR injury. At the same time, Boucherat et al. [51] found that the proliferation and migration of PAH PASMCs could be affected by the pharmacology of HDAC6, tubastatin A, ACY‐775, ACY‐241, or siHDAC6, which increased the resistance to apoptosis. In addition, blocking HDAC6 reduced abnormally high right ventricular systolic and pulmonary artery pressure, as well as right ventricular and vascular remodeling, thereby improving sugar/hypoxia and medium‐chain triglyceride‐induced PAH. Renal fibrosis was inhibited by tubastatin A regulating epigenetic histone modifications and SMAD3‐dependent fibrosis genes [40]. More recently, tubastatin A was shown to be irreplaceable in a canine model of AF [11]. Dogs treated with tubastatin A were protected from disrupted α‐tubulin homeostasis and electrical remodeling caused by atrial tachycardia, thereby improving cellular Ca2+ processing and contractile function, and effectively counteracting AF progression [9, 11]. Additionally, AF‐induced deacetylation was prevented by HDAC6 inhibition, along with subsequent depolymerization and degradation of α‐tubulin degradation by calpain 6. In this way, tubacin ensured protein stabilization and contractile function of α‐tubulin in AF experimental cardiomyocytes [52]. The current research status of inhibitors related to cardiovascular disease is summarized in Table 2.

**Table 2:** A Summary of HDAC6 inhibitors involved in cardiovascular disease.

Inhibitor	Inhibitor structure	Types of cardiovascular disease	Regulatory mechanism	References
ACY‐1215	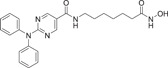	Myocardial infarction	Regulating HIF‐1α	[34]
ACY‐775	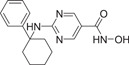	Pulmonary hypertension	Regulating proliferation and migration of PAH PASMCs	[51]
Trichostatin A	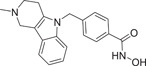	Myocardial infarction; pulmonary hypertension; arrhythmia	Regulating epigenetic histone modification; α‐tubulins; SMAD3‐dependent fibrosis genes	[9, 11, 40, 47]
Tubacin	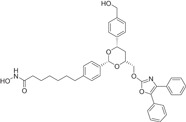	Arrhythmia	Regulating TGF‐β1; HSPB1	[52]
Vorinostat	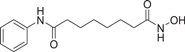	Myocardial infarction; myocardial fibrosis; arrhythmia; atherosclerosis	Regulating NOX, TNF‐α; LVEDP; metabolic effects; inflammatory factors	[11, 37, 68, 69]
Trichostatin A	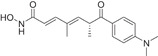	Myocardial infarction cardiac fibrosis	Regulating expression of actin, collagen and fibronectin of α‐smooth muscle; histone acetylation	[11, 38, 70]
Trichostatin A	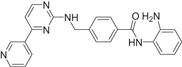	Myocardial infarction cardiac fibrosis	Regulating expression of actin, collagen and fibronectin of α‐smooth muscle	[11]
Entinostat	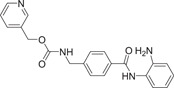	Cardiac fibrosis	Regulating fibroblasts and myofibroblasts	[11]
Sodium butyrate	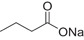	Cardiac hypertrophy	Regulating angiotensin II; α‐tubulins and β‐myosin	[9, 11]
Valproic acid	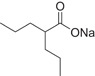	Heart disease; pulmonary hypertension; cardiac hypertrophy; myocardial infarction	Regulating expression of COX2/PGE2; ANP and pERK phosphorylated ERK can be stimulated by Ang II	[9, 60]

## 5 SUMMARY AND FUTURE PROSPECTS

Many studies have shown that HDAC6 plays a crucial regulatory role in the development of cardiovascular disease. This occurs mainly by the regulation of HDAC6‐mediated acetylation of hERG, Prdx1, phosphorylated SMAD2/SMAD3, ERK1/2, HSP signaling pathways, and controlling signal transduction via RASSF1A, TGF‐β1, MeCP_2_, and others. Therefore, HDAC6 has an important influence on the improvement and treatment of cardiovascular diseases. Although HDAC6 plays a regulatory role in the physiological and pathological processes of cardiovascular disease, there are still many questions that deserve further investigation. Many in vivo roles of HDAC6 and the mechanisms of its regulation remain unclear [15]. Also, a considerable number of interactions between HDAC6 and other gene regulators remain indistinct [15]. In addition, few HDAC6 inhibitors have been designed and synthesized to treat cardiovascular disease from the perspective of pharmaceutical chemistry using computer‐aided drug design and structure‐based drug design strategy [37]. It is worth noting that clinical trials of HDAC inhibitors started many years ago. Since then, HDAC6 has become a good therapeutic target of many diseases include cardiovascular disease. Ideally, HDAC6 displays a unique structure and cellular localization, as well as diverse substrates and a wider range of biological functions than other HDAC isoforms. We found that HDAC6 inhibitors in combination with other agents have promising prospects for the treatment of cardiovascular disease. Honokiol improves Ang II‐induced hypertension and endothelial dysfunction by inhibiting HDAC6‐mediated degradation of cystathionine γ‐lyase [47]. Meanwhile, HDAC6 structure and diverse substrates serve as ideal targets on which dual‐targeted inhibitors can be designed. Yang et al. [24] found that HDAC6 combined with LSD1 (as lysine specific histone demethylase 1) was an excellent target to develop novel potent dual‐targeted inhibitors, with their extensive interconnectedness in various human diseases [76, 77]. For example, Bulut et al. [78] have shown that Dox toxicity can be reduced by parallel inhibition of HDAC6 and LSD1. Thereafter, we observed that a combination of chemotherapies and HDAC6 inhibitors became a “hot topic” in the treatment of cardiovascular disease. Song and colleagues [1] first discovered that HDAC6 inhibition protected cardiomyocytes against Dox‐induced injury without influencing the effect of Dox on inhibiting MDA‐MB‐231 subcutaneous tumor growth in vitro and in vivo. Moreover, α‐tubulin hyperacetylation may account for protection against Dox‐induced damage to mitochondria and autophagy [1]. Bagchi et al. [64] also demonstrated that Dox‐induced cardiotoxicity in mouse heart could be protected by tubastatin A; this included left ventricular dysfunction, myocardial fibrotopenia, and cytoplasmic vacuolation. We also found a combination of HDAC6 inhibition with immune factors for cardiovascular disease therapy [79]. Recent studies have shown that regulatory cells [50] (CD4+, CD25+, FOXP3+) can prevent vascular injury and resist pulmonary hypertension. Baicalin increased Tregs by downregulating HDAC9 and HDAC6 expression, which inhibited the development of AS, and ultimately promoted acetylation of FOXP3 [80]. In brief, HDAC6 is a new field in the diagnosis, treatment, and related research of cardiovascular disease.

Together, the relationship between HDAC6 and cardiovascular disease needs further investigation. Designing and synthesizing HDAC6 inhibitors in combination with other agents may be an excellent strategy for tumor cardiology in the future.

## AUTHOR CONTRIBUTIONS

**Ya‐Xi Wu**: Investigation (equal); writing—original draft (lead). **Bing‐Qian Li**: Investigation (equal). **Xiao‐Qian Yu**: Investigation (equal). **Yu‐Lin Liu**: Investigation (equal). **Rui‐Hao Chui**: Investigation (equal). **Kai Sun**: Funding acquisition (lead); investigation (lead); resources (lead); writing—review & editing (equal). **Dian‐Guang Geng**: Conceptualization (equal); funding acquisition (lead); investigation (equal); resources (lead). **Li‐Ying Ma**: Conceptualization (equal); funding acquisition (lead); investigation (equal), resources (lead); writing–review & editing (lead).

## CONFLICT OF INTEREST STATEMENT

The authors declare no conflict of interest.

## ETHICS STATEMENT

Not applicable.

## INFORMED CONSENT

Not applicable.

## Data Availability

Current studies indicate that HDAC6 can promote the occurrence and development of related tumors and cardiovascular diseases, and inhibiting its activity can effectively inhibit tumor progression and cardiovascular damage, which is a regulatory mechanism for the association between tumor and cardiovascular diseases.
